# New products

**DOI:** 10.1155/S1463924688000434

**Published:** 1988


Journal of Automatic Chemistry Vol. 10, No. 4 (October–December 1988), pp. 201–209

### New products

### Chemical modelling

U-Microcomputers was founded in 1978 by Dr Bill Unsworth – a chemist with broad experience of the specialist needs of the analytical chemist through employment with major manufacturers of scientific instrumentation. The company became a dealer for Apple Computers in 1979 and in 1983 U-Micro became one of the very first IBM Business centres in the UK. By consolidating the company as a supplier of 'off-the-shelf' products, it was possible to search out new and profitable applications of microcomputers in the scientific field.

U-Micro firstly developed a wide range of specialized interfacing and add-on products, initially for the Apple II and later for the IBM range, to enable scientists to connect their computers to many other scientific instruments. These 'add-ons' allowed the utilization of microcomputers in fields where previously more powerful and expensive mini-computers had to be used.

Building on this experience U-Micro launched their own technical computer system 'U-MAN' based on the 68000 microprocessors, which, unlike those used in standard (IBM compatible) microcomputers are 'multi-user'. A recent major order for such a system has seen a complete suite of 25 interlinked work-stations which have been utilized by UMIST to provide comprehensive high level programming training for the first year intake on their successful computer science course, at a fraction of the cost of alternative systems.

How do we get from 'scientific' computers to the design of new molecules? Purchasers of the U-Micro computer started to utilize the power of these relatively inexpensive systems to replace very expensive mini-computers and specialized graphics systems in designing novel chemical structures. An extensive period of collaboration followed, resulting in a specialized chemical modelling system with high resolution graphics – 'Chemmod'.

U-Micro Chemical Modelling systems are now in use worldwide, solving problems that were either impossible or extremely time consuming a few years ago. One such system is playing a major role in the development of anti-cancer drugs at the Paterson Cancer Research Institute, Christie Hospital in Manchester.

U-Micro are currently developing the use of the Inmos Transputer chips to provide orders of magnitude improvement in scientific 'number crunching' in all their products. These products will also drastically cut the time needed for energy calculations on complex structures – of many thousands of atoms – a move which is exciting a large number of multi-national pharmaceutical companies.

*Details about the company and its products from U-Microcomputers Ltd, 12 Chetham Court, Winwick Quay, Warrington, Cheshire WA2 8RF, UK.*

### DNA amplification system

With the new Perkin-Elmer Cetus DNA Amplification System, molecular biologists can now produce microgram amounts of DNA from picogram amounts of starting material using the Polymerase Chain Reaction (PCR) technique. The PCR technique provides primer-mediated enzymatic amplification of specific genomic sequences. It is a simple molecular biology method designed by scientists at Cetus Corporation to significantly amplify *in vitro* both genomic and cloned DNA sequences. A target DNA sequence can be amplified *in vitro* at least 100 000-fold in a few hours.

The DNA Amplification System consists of the Gene-Amp DNA Reagent Kit and the DNA Thermal Cycler, a microprocessor-controlled temperature cycling instrument, designed to simplify and optimize the PCR technique. The Gene-Amp kit contains optimized reagents and an easy-to-follow experimental protocol to perform the highly selective PCR technique. By using a thermostable enzyme, Taq Polymerase, the Gene-Amp kit avoids the cumbersome task of adding fresh enzyme after each amplification cycle. The kit contains sufficient reagents to perform 100 individual assays – Taq Polymerase, dinucleotide triphosphates, buffers and a control template with matching primers to verify kit performance.

The DNA Thermal Cycler is a highly sophisticated, fully programmable, yet easy-to-use temperature cycling instrument specifically designed to automate the PCR technique. Each amplification cycle consists of three processes: template denaturation, primer annealing and primer mediated extension, which occur at specific temperatures. The rapid temperature changes and incubation times are programmed and controlled by the DNA Thermal Cycler, enhancing the user's ability to control and optimize the PCR technique to achieve maximum amplification efficiency.

*Details from Perkin-Elmer (p. 202).*

### LIMS 2000 software available on digital computers

Perkin-Elmer's LIMS 2000 Laboratory Information Management Software is now available on Digital Equipment Company's full line of VAX computers.

The LIMS 200VX system of data collection, manipulation, storage and management enables chemists to extract more information from data. They can investigate how samples change over time, compare the results from one sample with another and examine the results from different analytical techniques. Historical data is easily recalled, so that trends can be compared. A significant benefit is the speed with which reports, ranging from short status summaries to complete reviews of analytical results can be produced. The built-in audit trail of LIMS 2000VX assures accountability of analytical data to government agencies and helps laboratories comply with Good Laboratory Practice (GLP) regulations.

LIMS 2000VX data-base design is easily tailored to individual laboratory requirements, and includes applications software for sample entry, results entry and system management and support services including comprehensive training and custom programming, as well as an SQL query language and report generation software.

LIMS 2000VX meets Digital's ILA (Integrated Laboratory Automation) standards, which establish criteria for communications, data management and data integration of computer-based laboratory products, using DECnet software to ensure that products from Digital and laboratory instrument and software vendors can communicate and share data.

*Further information from Perkin-Elmer (see right).*

### Sodium, potassium and chloride ISE analysis in 35 s

From a sample of only 65 μl, the Ciba Corning 644 ISE analyser will provide a stat result for sodium, potassium and chloride in less than 35 s. This level of analytical efficiency is also achieved in batch analysis as 60 samples can be automatically processed in one hour.

The 644 employs the same advanced ion selective electrode technology as the 614 sodium/potassium analyser and the 634 ionized calcium and pH analyser. An alphanumeric display guides the operator through all sequences via an interactive dialogue using two yes/no answer controls. All automatic functions, including calibration frequency, sample detection, maintenance routines and flushing cycles, are under direct control of the microprocessor. Once the whole blood, plasma, serum or diluted urine has been aspirated, it is positioned in the measuring chamber, where twin optical detectors confirm correct positioning and integrity. Samples containing bubbles can be repositioned manually and then measured as normal.

The analyser is suited for the acute care laboratory and the emergency or paediatric section of a clinical chemistry department. When combined with the 604 Autosampler, the 644 attains high productivity and low cost per test as the primary electrolyte analyser in the small laboratory.

*Further details from Ciba Corning Diagnostics Ltd, Halstead, Essex CO9 2DX, UK. Tel.: 0787 472461.*

### Low-cost HPLC packages

Perkin-Elmer have announced the availability of low-cost packages for liquid chromatography. Based around isocratic solvent delivery these packages include either diode array or conventional UV detection and data processing. Prices for the complete system including injector valve, bracket and column vary from less than £9000 to £13 210 for the diode array system.

*For further information, contact Perkin-Elmer Ltd, Post Office Lane, Beaconsfield, Buckinghamshire HP9 1QA, UK. Tel.: 04946 6161.*

### Diode array detection system

Philips Analytical has launched its new PU6003 diode array system for liquid chromatography. Designed for use with the PU4021 or PU4120 diode array detector, the system uses Chromascan II software.

Chromascan II operates in the IBM environment and runs under multitasking windows. A range of peak purity tests are provided to assure total confidence in LC method validation. Additionally, a comprehensive set of data manipulation algorithms is available.

*More information from Philips Scientific, York Street, Cambridge CB1 2PX, UK. Tel.: 0223 358866.*

### Literature from VG Isogas

Two brochures are now available from VG Isogas, describing the PRISM Series II stable isotope ratio mass spectrometer, and a range of sample preparation systems.

The application areas of stable isotope ratio mass spectrometry include geology, oceanography, petroleum exploration, agronomy and medical research.

The PRISM Series II is a high performance instrument controlled by the IBM PS/2 microcomputer.

The sample preparation systems described are compatible with each of the stable isotope mass spectrometers produced by VG Isogas. They provide the user with methods, often fully automated, for preparing and purifying sample materials prior to introduction into the mass spectrometer. The brochure describes over 12 different sample preparation systems which cover all types of application areas.

*Details from Dr Nigel Crossley, VG Isogas Ltd, Aston Way, Middlewich, Cheshire CW10 0HT, UK. Tel.: 0606 845151.*

### Pulsed amperometric electrochemical detection

The M400 electrochemical detector from Jones Chromatography, offers the liquid chromatographer a novel analytical technique. Using conventional amperometric LC-EC at a constant potential it is not possible to detect many organic compounds with Pt or Au electrodes.

Compounds such as carbohydrates, amino-acids and non-aromatic alcohols can, however, be satisfactorily detected using a pulsed potential waveform. The electrode is sequentially subjected to three distinct potentials which respectively prime the electrode, perform the oxidation reaction, and clean the electrode. A current is measured only during the intermediate oxidation stage.

*For further details and applications contact Jones Chromatography Ltd, New Road, Hengoed, Mid Glamorgan CF8 8AU, UK.*

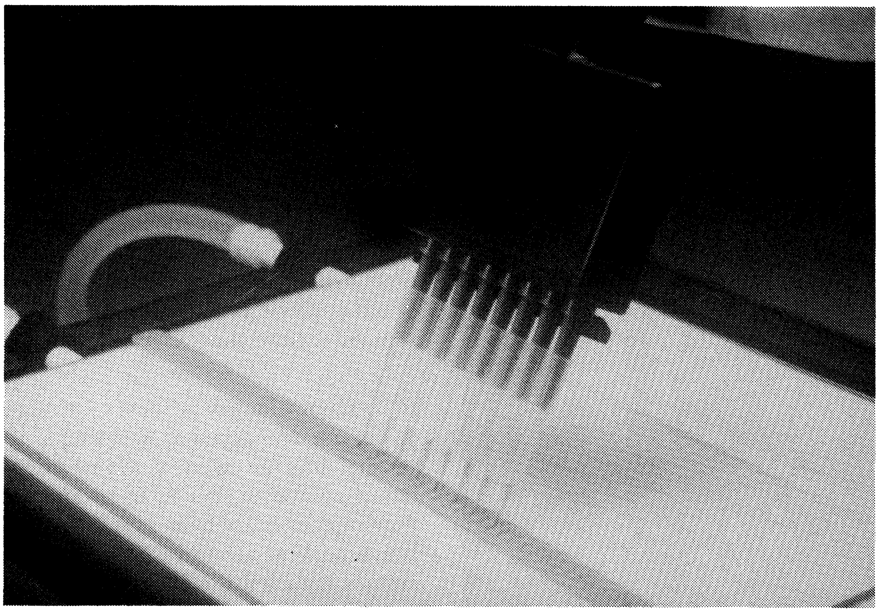

*Up to 52 samples of 20 μl can be applied to one Immobiline Isoelectric focusing gel using a new applicator strip from Pharmacia LKB Biotechnology AB. The well spacing of the strips is such that most types of multipipette systems can be used improving sample handling and convenience. Based on an idea of Dr W. Pflug of the Kriminaltechnisches Institut, Landeskriminalamt Baden-Würtemberg, Stuttgart, the silicone rubber strip overcomes problems associated with other gel loading methods. Details from Pharmacia LKB, Biotechnology AB, Box 305, S-161 26 Bromma, Sweden.*

### Ferranti-Shirley viscometers

In response to continuing demand for its viscometers, Ferranti Instrumentation is extending its spares and repair service in support of units already in the field. All spares and servicing will be carried out from the Commercial Instrument Divisions Laboratories, at Moston, Manchester.

Designed for recording the flow behaviour characteristics of a wide range of fluids from solvents through to highly viscous liquids, the Ferranti-Shirley viscometer uses a rigid cone to plate measuring head geometry, ensuring high accuracy over a wide range of samples, giving dynamic results which may be plotted graphically enabling analysis of shear-dependent and time-dependent flow behaviour.

*Details from Ferranti Instrumentation Ltd, Commercial Instruments, St Mary's Road, Moston, Manchester M10 0BE, UK. Tel.: 061 681 2071.*

### Manual and automatic titration directly in COD digestion tubes

Tall, narrow tubes for digestion blocks are commonly used for the chemical digestion of samples to be analyzed for Chemical Oxygen Demand. Up till now, the subsequent titration of the excess dichromate solution has had to be performed in a glass beaker.

With the ASPI 1000 Combined Redox Electrode, a potentiometric titration can be performed directly in the narrow digestion tube as the effective length of the electrode is 175 mm and the diameter 9·5 mm. By titrating in the tubes, decanting of the concentrated acid-mixture is avoided, and so the precision of the analysis is increased and hazards minimized.

The redox sensing element is platinum, whereas the reference cell is mercury/mercurous-sulphate. The potassium-sulphate electrolyte is chosen rather than a cell with potassium-chloride which would give an unstable potential caused by reaction with the silver catalyser used for complete digestion of the sample.

*Single samples*

For analysing for COD (Chemical Oxygen Demand) in small series, a *dedicated sample stand* is recommended. The stand will safely get the Combined Redox Electrode, a titrant delivery tip and a stirrer propeller down into the digestion tube. The propeller secures effective stirring in the tall, narrow tube, and fiddling with magnets is avoided.

*Automatic titration* can be performed, for instance by using the DTS812 Multi Titration System which will, in a few minutes, accurately detect the drop in potential, calculate the result of the back-titration, and print out the COD result in mg/l. An application note with statistics for a wide range of COD-values is available.

*For further information contact Radiometer Analytical A/S, 49, Krogshojvej, DK 2880 Bagsvaerd, Denmark. Tel.: 45 1 696311.*

### Chiral columns

Many compounds, among them pharmaceuticals, exhibit different reactions and different toxicological effects depending on their chiral forms. The thalidomide tragedy is the best known example of the unexpected and sometimes disastrous results that chiral differences can cause.

The development of special chiral columns allows the separation of milligram quantities so that the different enantiomers of a compound may be studied.

Hewlett-Packard now produce chiral columns for the separation of organic macromolecules, so that the 'mirror image' structural differences characteristic of the different enantiomers may be identified.

HP's chiral columns are available from local HP Supplies Offices, and may be ordered through the supplies hotline available in most European countries.

*Enquiries to Tina Green, Analytical Products Group, Hewlett-Packard Ltd, Miller House, The Ring, Bracknell, Berkshire RG12 1XN, UK. Tel.: 0344 424898.*

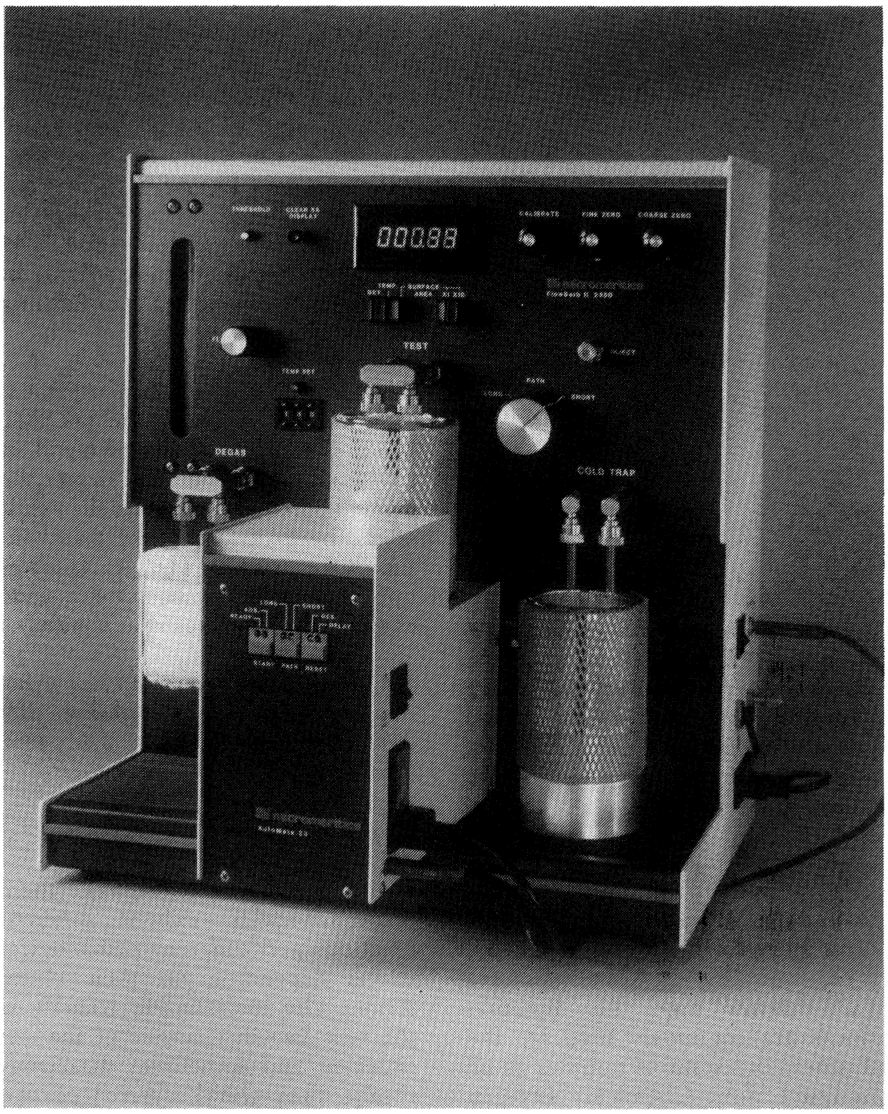

*The AutoMate 23, from Micromeritics, is a compact robotic device that makes running surface area analyses on a FlowSorb Analyzer easier. FlowSorb users can start a surface area analysis and then walk away. Tasks the AutoMate performs include: triggering the adsorption process, that is, elevating the $LN_2$ cold bath: initiating the desorption process, that is, removing the $LN_2$ cold bath and rapidly warming the sample; and alerting the operator with audible and visual signals that the surface area result is ready. More information from Micromeritics, One Micromeritics Drive, Norcross, Georgia 30093-1877, USA.*

### System Gold solvent delivery

The stand-alone System Gold delivery modules from Beckman (the Model 116 isocratic and Model 126 gradient) offer the highest flow specifications available from a liquid chromatograph.

Great flexibility of application is offered by the modules. A flow rate range of 1 μl to 10 ml/min can be obtained from each module (1 μl to 20 ml/min isocratic for the Model 126), with reproducibility of ±0·1% and maximum operating pressure of 6000 psi. Each module provides complete time programmability of flow rate, solvent selection facilities (four isocratic and eight gradient are standard) and six standard contact closures. A five-year seal replacement warranty is an added benefit.

Automatic solvent compressibility correction at the pump head is a unique feature of these modules which eliminates the need for solvent degassing in the majority of HPLC applications.

*Beckman, Progress Road, Sands Industrial Estate, High Wycombe, Buckinghamshire, UK. Tel.: 0494 41181.*

### FIA

Flow injection analysis is very much a topic of the moment, with more than 1000 academic papers having been published on the technique in recent years. Until now, however, the application of flow injection analysis has been limited by the high cost of establishing an analysis facility with a basic piece of equipment costing typically more than £15 000.

A revolutionary new design approach developed at Reading University has allowed a Cambridge-based company, WPA, to produce a flow injection analysis system which is available at around £3000: the new system is called the Heli-flow.

*Further information from Michael Holland, Smye Holland Associates, 63 Park Road, Peterborough PE1 2TN, UK. Tel.: 0733 64906.*

### Chemical analyses for oil users

Efficiency, economy or safety will always be improved by periodic monitoring of petroleum products used in fuelling or lubricating engines or machinery. Irreparable damage is easily sustained by machinery where lubricants have become too acid. With this in mind, Radiometer's application chemists have prepared a set of six chemical analyses which are available, free of charge, to industrial company laboratories handling petroleum products.

All six procedures are based on potentiometric titrations with Radiometer's 'TitraLab'. The procedures are: total acid number; total base number; bromine number; mercaptan determination (all to ASTM and/or ISO standards), and $H_2O$ determination in petroleum products by Karl Fischer titration.

*Radiometer's petroleum product analysis procedures have been described published in a free 28-page booklet, which is available from the Analytical Division, Radiometer Ltd, The Manor, Manor Royal, Crawley, West Sussex RH10 2PY, UK. Tel.: 0293 517599.*

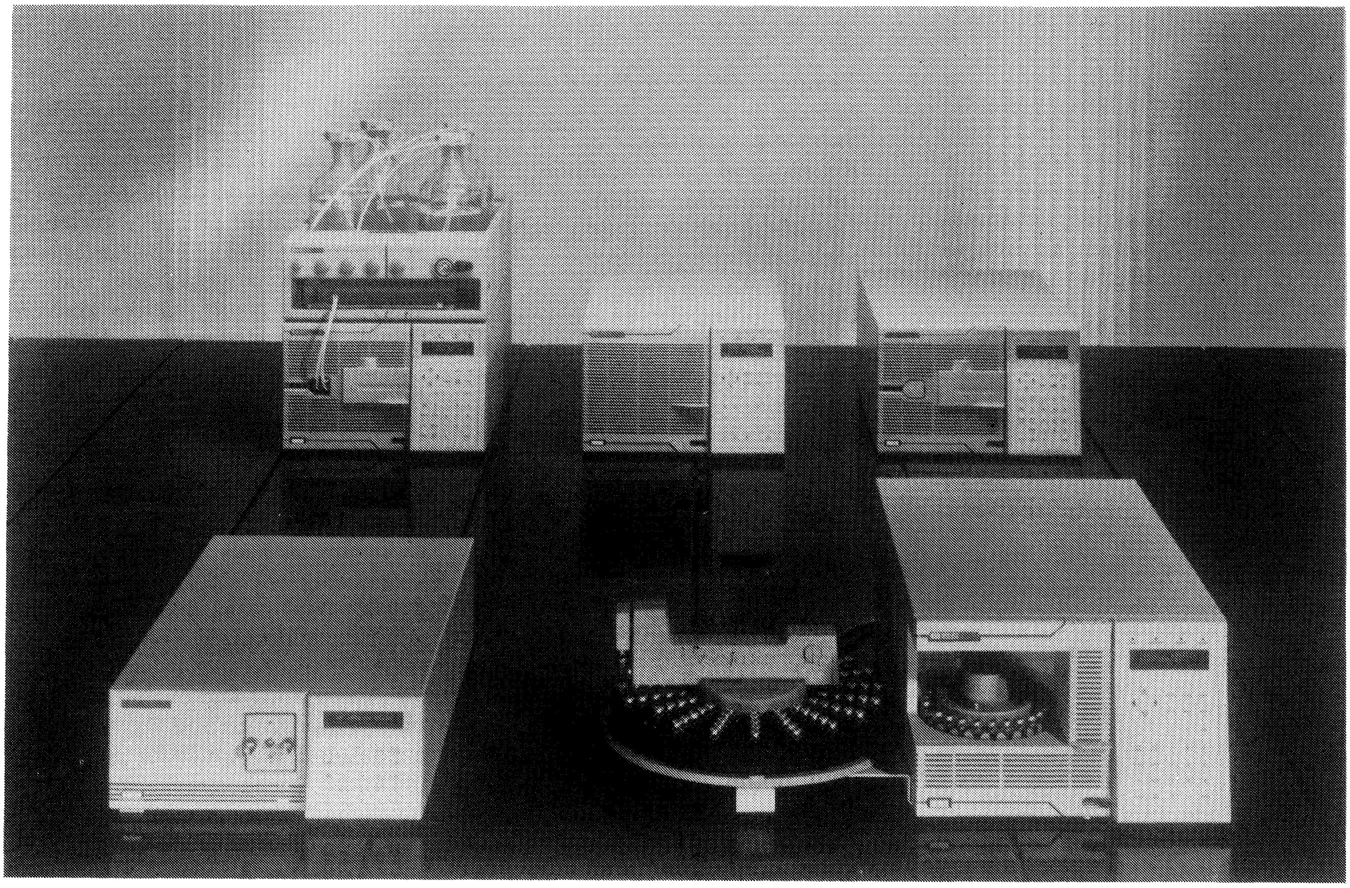

*Each one of the five new HP 1050 series HPLC modules from Hewlett-Packard Company is a reliable partner to any HPLC system. Left to right, rear: quaternary pumping system, multiple-wavelength detector and isocratic pump. Left to right, front: variable-wavelength detector and autosampler.*

### HPLC modules

Hewlett-Packard Company has introduced the HP 1050 series of HPLC modules, allowing chemists to upgrade current modular HPLC systems to HP modules one at a time. The five modules have been designed to meet demanding requirements for accuracy, precision, sensitivity and reliability.

Designed with standardized packaging, user-interface and module-to-module communication, the HP 1050 series modules are US list priced from \$5600 to \$12 400.

The five members of the HP 1050 series are:

*Isocratic pump* – the isocratic pump uses a serial dual-piston approach to solvent delivery, with variable-stroke volume to achieve flow stability across a wide flow range.

*Quaternary pumping system* – the same single pump is the basis for the quaternary pumping system. HP's own high-speed proportioning valve mixes the mobile phase at low pressure, which provides gradient capability for up to four solvents. A separate solvent cabinet degasses and filters the solvents.

*Variable-wavelength detector* – the programmable variable-wavelength detector features high sensitivity, wavelength-switching and stop-flow scanning. The detector's interchangeable cells meet the requirements of many different HPLC application areas.

*Multiple-wavelength detector* – the multiple-wavelength detector uses diode-array technology for dual-wavelength monitoring and scanning without stopping the flow.

*Autosampler* – the autosampler's precision injection mechanism features programmable injection volume up to 400 μl. Microprocessor control permits calibration routines independent of sample position and variable sample capacity (a 21-sample tray is included; a 100-sample tray is optional).

The five modules are moved easily from one liquid chromatograph (LC) site to another.

*Full information from Hewlett-Packard (as below).*

### New brochure on 'chromatography worksystem'

A full-colour brochure from Hewlett-Packard describes in detail the HP 3359 'chromatography worksystem', which combines workstation simplicity with the data management capability of a laboratory automation system. Hewlett-Packard now offer free telephone support for a three-month period to purchasers of this system, providing an immediate response to any software problems that may arise.

*Copies from Analytical Products Group, Hewlett-Packard Ltd, Miller House, The Ring, Bracknell, Berkshire RG12 1XN, UK. Tel.: 0344 424898.*

### Wyeth announce developments in R & D

Wyeth Laboratories have announced that they are investing in a major development at their Institute of Medical Research in Taplow, Berkshire, UK. The Institute, which was opened in 1965, forms an integral part of Wyeth's world-wide research facilities.

The developments, which will cost some £3·5 M, will in particular extend accommodation for behavioural pharmacology and physical chemistry and also provide an advanced computer controlled R & D environment.

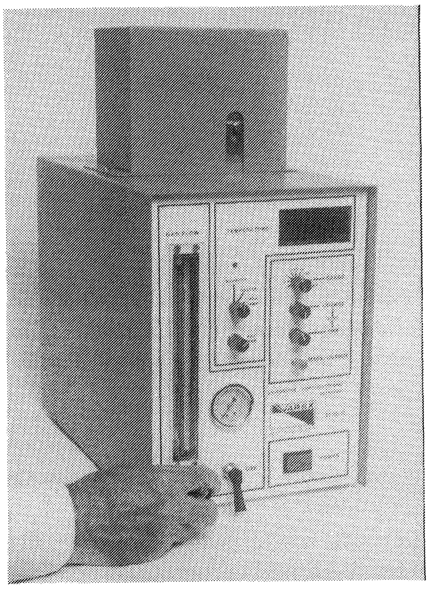

*The latest revision to the Varex Evaporative Light Scattering Detector ELSDII for HPLC/GPC now has a simplified nebulizing gas flow which enhances the solvent evaporation principle of the ELSD. Responding to virtually all solutes, the detector has a more powerful and precise nebulizer heater enabling efficient evaporation of aqueous solvents and operates efficiently with multiple solvent gradients. A descriptive brochure is available from Varex, 12221 Parklawn Drive, Rockville, Maryland 20852, USA. Tel.: 301 984 7760.*

### Video

An 11-minute video describing the Zymate II Laboratory Automation System for analysing drugs and metabolites in biological fluids is available for a 15-day trial period. The system automates the following steps:

- Temperature-controllable sample storage
- Sample aliquotting
- Reagent and buffer addition
- Internal standard addition
- Liquid–liquid extraction solvent addition
- Tumble mixing
- Centrifugation for phase separation
- Liquid–liquid extraction sample collection
- Evaporation and reconstitution
- Solid phase extraction
- Sample filtration
- HPLC injection or sample storage.

*For more information contact John Helfrich, Zymark Center, Hopkinton, Massachusetts 01748, USA. Tel.: 617 435 9501.*

### SERC funding for PS Analytical

PS Analytical has many close ties with industrial and academic partners. Two current examples of successful co-operation are the Valve Switching Data Transfer Interface, which was developed jointly with Glaxo Operations Ltd at Barnard Castle, and the Ebdon High Solids Nebulizer, designed with Plymouth Polytechnic.

The need to continue this approach and to build on the skills of PS Analytical in the design of automated systems is well recognized. Combining different philosophies to solve difficult analytical situations in a simple and easy-to-use manner, PS Analytical and Professor L. Ebdon's Group, at Plymouth Polytechnic, have formed a joint research programme. Funded by the Science and Engineering Research Council, the programme will offer the opportunity of academic training, leading to a PhD degree by thesis, and a strong interaction with a developing instrumentation company.

*Suitably qualified persons interested in working in such a programme are invited to contact either Professor Ebdon (Department of Environmental Sciences, Plymouth Polytechnic, Drake Circus, Plymouth PL4 8AA), or Dr P. B. Stockwell (PS Analytical Ltd, Arthur House, Cray Avenue, Orpington, Kent BR5 3TR). A science graduate with a good honours degree and an interest in new approaches to automation would make a suitable candidate.*

### Multiple wavelength universal UV detector

Perkin-Elmer is a new high sensitivity dual wavelength programmable UV detector for HPLC, the LC 135, which is based on diode array technology, provides precise wavelength selection and instantaneous wavelength change without moving parts. As a result, unlike conventional monochromator detectors, wavelength accuracy and reproducibility are not compromised due to mechanical wear.

The LC-135 can be used in a universal UV mode, detecting all UV absorbing species regardless of the monitoring wavelength for method development or as a validation aid. With the addition of a printer plotter the LC-135 becomes a 'smart detector', calculating and outputting peak purity, ratiograms and wavelength of maximum absorbance for peak identification, peak tracking, or for wavelength switching programs.

*For further information contact Perkin-Elmer Ltd, Post Office Lane, Beaconsfield, Buckinghamshire HP9 1QA, UK. Tel.: 04946 6161.*

### High performance GC/FT-IR interface

A gas Chromatography/FT-IR interface has been announced by Perkin-Elmer for their 1700-X Series FT-IR spectrometers and 8000 Series gas Chromatographs. The interface is configured 'in-line' in an auxiliary sampling compartment, leaving the primary compartment free for routine analyses.

A high temperature low volume transfer line and light-pipe assembly is matched to a cooled MCT detector, giving identifiable spectra at low nanogram levels.

Full injection-to-identification automated GC/FT-IR methods may be performed using the Perkin-Elmer Model 7700 Professional Computer.

*For further information contact Perkin-Elmer Ltd, Post Office Lane, Beaconsfield, Buckinghamshire HP9 1QA, UK. Tel.: 04946 6161.*

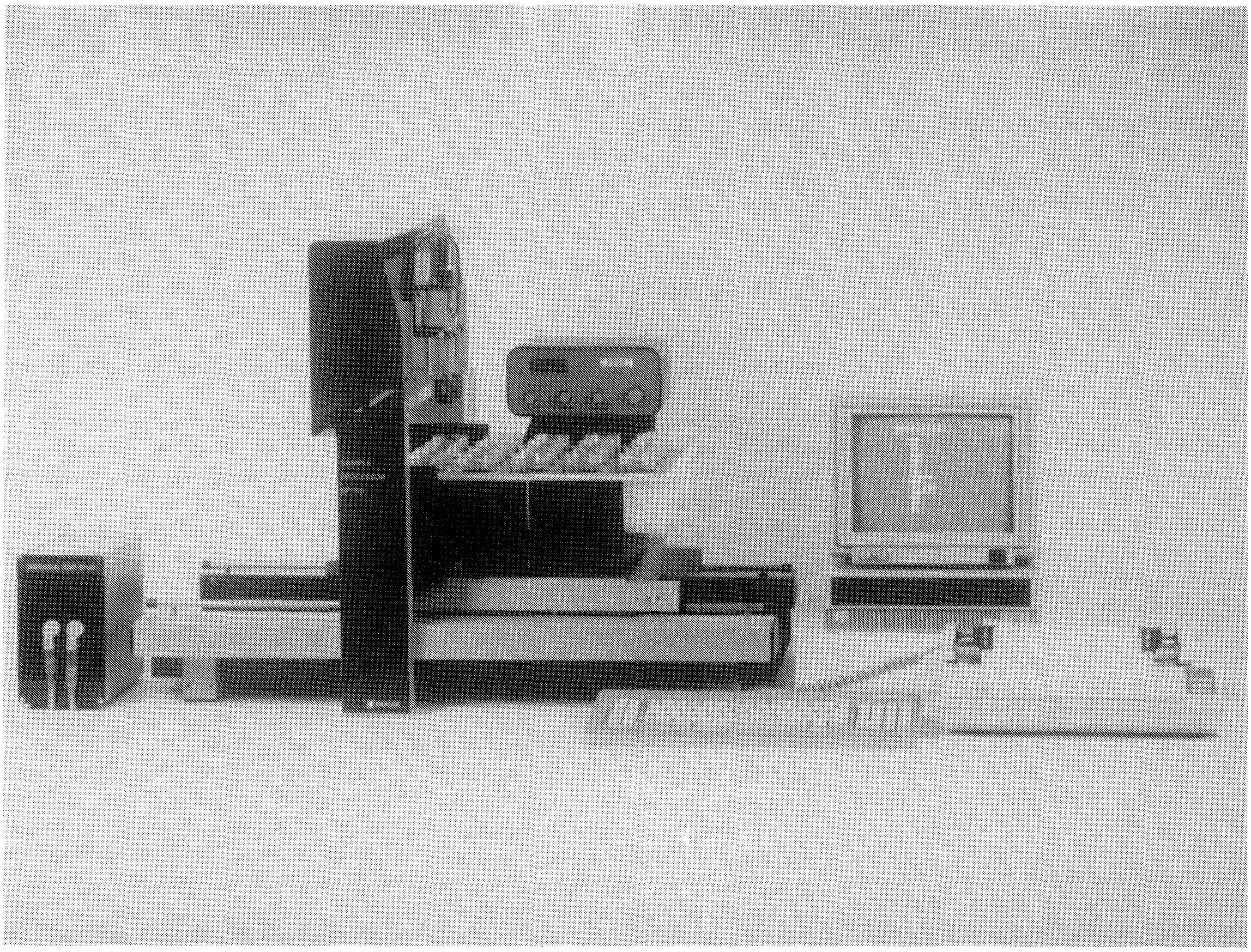

*The SP100 is a fully automated system for soil pH. The system offers total automation and control of the water addition, stirring, pH measurement, calibration, calculation, and data manipulation. The system is standard for one rack. Expandable up to four racks of 20 samples each, enabling overnight soil pH analysis. Full information from L. Plevier, Skalar Analytical BV, Spinveld 62, 4815 HT Breda, The Netherlands.*

### The Macintosh analogue connection

The Strawberry Tree Analogue Connection range of data acquisition boards and software recently introduced by the UK by Adept Scientific, is now also available for Apple Macintosh SE and Macintosh II computers.

High resolution, 8 or 16 channel boards offer up to 16 bit conversion for precise data acquisition. Fixed gain ranges or autoranging can be independently selected on each channel giving a dynamic range of 1 $\mu$V to 10 V.

Virtually any combination of transducers, including resistance temperature detectors (RTDs), thermocouples, strain gauges and load cells, may be interfaced to the same board.

Automatic procedures eliminate tedious calibration and switch-setting routines. A factory-calibrated reference source re-calibrates the board automatically during operation, maintaining zero and ensuring full-scale accuracy and stability. Self-diagnostic software identifies which board is in use, checks its functions and reports any failure.

Data acquisition, logging, control and display capabilities are offered by the Analogue Connection Workbench software. Icons are dragged to a worksheet from a graphical menu and connected together by drawing lines on screen. Icons are available to control analogue and digital input and output, alarms, graphics, calculations and data logging. Over 400 icons may be included in a single set-up and complete set-ups can be stored and retrieved from disk.

QuickLog, a simpler version of the Workbench package which can be upgraded to full Workbench specification when required, is included free of charge with Strawberry Tree's Macintosh boards, as is the Analogue Connection Development System.

Logged data may be imported into spreadsheets, data-bases and analysis software packages, including Excel and Parameter Manager Plus.

Strawberry Tree boards are covered by a two-year hardware warranty that even covers the self-calibration feature. Full customer service and support is provided by Adept Scientific.

*Further information from Adept Scientific at 3 Letchworth Business Centre, Avenue One, Letchworth, Hertfordshire SG6 2HB. Tel.: 0462 480055.*

### Techogel HPLC columns for protein/peptide analysis

The Techogel range of 300 Å and 500 Å silica and bonded phase columns is intended for the separation of large molecules, such as proteins, peptides, enzymes, and other biopolymers. Other 300 Å phases commercially available tend to collapse at high pressures, meaning that separation efficiencies are low and column lifetimes are short. The rigid 300 Å and 500 Å silicas used in the Techogel range eliminate these problems.

The 300 Å range is available in 5 and 10 micron particle sizes, and 3 μm phases are also offered. Phases include silicas, C4, C8 and C18.

*Further information from HPLC Technology Ltd, Wellington House, Waterloo Street West, Macclesfield, Cheshire SK11 6PJ, UK. Tel.: 0625 613848.*

### Microplate reader

The Labtec 2001 is being promoted as the most technically advanced photometer and data reduction instrument available for microplate reading. It is capable of performing colorimetric, nephelometric and agglutination assays without instrument modification. It also allows simultaneous multiple wavelength measurements for research and special clinical applications.

The Labtec offers five measurement modes for single or dual wavelength, agglutination readings, kinetic measurements with true 5-second reading intervals, multiple wavelength readings and as a totally remote controlled photometer.

*Literature from Denley Instruments Ltd, Natts Lane, Billingshurst, West Sussex RH14 9EY, UK. Tel.: 040381 3441.*

### 1 PS phase separator

Designed to separate aqueous and solvent phases, Whatman 1 PS is a high grade filter paper impregnated with a stabilized silicone which renders it hydrophobic, retaining the aqueous phase and passing the solvent phase through. In many applications it can replace the use of separating funnels.

A unique advantage of the 1 PS method is that cut-off is automatic and complete just as soon as the solvent phase has passed through. No skilled operators are required, any number of separations can proceed together and staff involvement in routine separations is at a minimum.

*Details from Whatman Ltd, Springfield Mill, Maidstone, Kent ME14 2LE, UK. Tel.: 0622 61681.*

### Clinical chemistry tests

Whether screening diabetics or checking cholesterol levels, nearly all the recent growth in clinical chemistry testing in Europe stems from one small twist the Italian government threw into its health coverage last year, according to a new report.

*The European Market for Clinical Chemistry Diagnostic Reagents (No. E995)*, a 259-page analysis completed recently by Frost & Sullivan, says that the $448 million in these test agents sold in Europe in 1987 represented a 6·8% increase, before inflation, from the year before. However, 'this apparently healthy rate of increase is deceptive, since almost all of it has occurred in Italy as a direct result of a political decision to annul the system whereby out-patients paid 25% of the cost of diagnostic procedures.' The volume of diagnostic testing in Italy 'rocketed up immediately' after that move by the government, the study says, although it believes the gains of as much as 57% cited elsewhere are erroneous and says a 25% average increase in testing is closer to the mark. (A reintroduction of mandatory patient payments toward the cost of diagnostic tests is possible, but is a current source of government negotiations.)

Frost & Sullivan notes that, excluding the Italian situation, the clinical chemistry market is a mature and slow-growing one. It analyses the four main countries in depth – France, the UK, FR Germany, and Italy – and reports that these account for more than two-thirds of Europe's total, or $374 million in 1987.

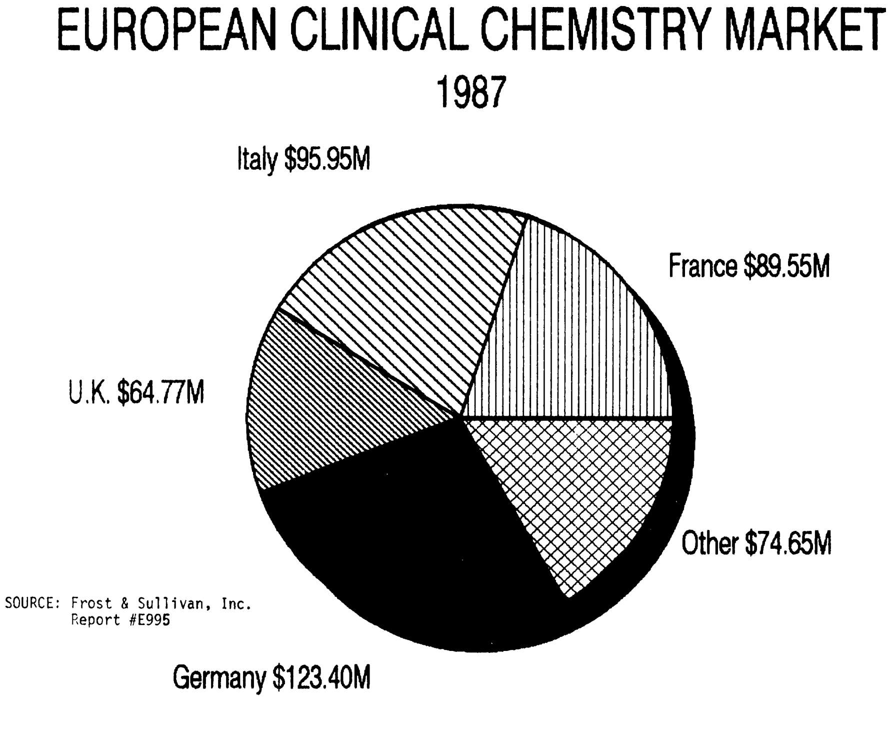

Overall, using constant dollars, the study predicts a clinical chemistry diagnostics market of $508 million in Europe by 1992, and $422 million in the four principal nations. The totals are dissected according to volume and value of various test types, location of testing (for example hospital versus private laboratory); supplier market shares in the different test sectors are given by country.

The substantial increase in the number of tests carried out in the doctor's group labs (*laborgemeinschaft*) and doctor's offices in West Germany is given credit as the second major factor in the Europe-wide increase in clinical chemistry – here, testing has been growing at a rate of 9·5% a year. However, politics will again intrude on the market, as a review of the current reimbursement system by the government will result in an anticipated 5% fall in sales volume of clinical chemistry reagents in 1988, though increases in control sera usage will partially offset this. (Control sera tests in particular will rise 8% to 10% a year in the forecast period; control sera is used as a means to check accuracy and assure standardized results in testing.)

Another feature of the West Germany market is that it is 'the best-developed in Europe for testing glycosylated haemoglobin levels in insulin-dependent diabetics; at over 1 million tests in 1987, almost 50% of this European testing was done there'. FR Germany, at $123·4 million in 1987, was the most important national sector for clinical chemistry tests; Frost & Sullivan expects that will hit $144·1 million by 1992.

France, which falls behind Italy and is the third-largest national market, at $89·6 million in 1987, should produce sales of $101·5 million by 1992. Within this, lipoprotein tests are becoming popular, gaining a steady 6% a year in number, while glycosylated haemoglobin checks are rising at twice that rate and the volume of control sera used is increasing by 4% to 5% a year. Private laboratories are important in France, as 35% to 38% of all testing is done outside the hospital.

In the UK, in contrast, 98% of clinical chemistry testing is done in hospitals. The lack of a doctor's office testing sector has effectively kept the diagnostic reagent market in check in the UK, with increases only on the order of 0·5% a year, and it seems likely to continue on the same course in the future. Clinical chemistry tests constituted a $64·8 million market in 1987 (or 41·5 million pounds), and are held likely to make up a $73·5 million one by 1992.

*For sales information, contact Customer Service, Frost & Sullivan, Ltd, Sullivan House, 4 Grosvenor Gardens, London SW1W 0DH, tel.: 01 730 3438; in the USA contact Customer Service, Frost & Sullivan Inc., 106 Fulton Street, New York, NY 10038; tel.: 212 233 1080.*

